# Administration of Adipose-Derived Stem Cells After the Onset of the Disease Does Not Lower the Levels of Inflammatory Cytokines IL1 and IL6 in a Rat Model of Necrotizing Enterocolitis

**DOI:** 10.3390/biomedicines12122897

**Published:** 2024-12-19

**Authors:** Marek Wolski, Tomasz Ciesielski, Kasper Buczma, Łukasz Fus, Agnieszka Girstun, Joanna Trzcińska-Danielewicz, Agnieszka Cudnoch-Jędrzejewska

**Affiliations:** 1 Department of Pediatric Surgery, Medical University of Warsaw, Zwirki i Wigury 63a, 02-091 Warsaw, Poland; 2 Laboratory of Centre for Preclinical Research, Chair and Department of Experimental and Clinical Physiology, Medical University of Warsaw, Banacha 1B, 02-097 Warsaw, Poland; tomasz.ciesielski@wum.edu.pl (T.C.); kasper.buczma@wum.edu.pl (K.B.); agnieszka.cudnoch-jedrzejewska@wum.edu.pl (A.C.-J.); 3 Department of Pathology, Medical University of Warsaw, Pawinskiego 7, 02-106 Warsaw, Poland; lukaszpiotrfus@gmail.com; 4 Department of Molecular Biology, Institute of Biochemistry, Faculty of Biology, University of Warsaw, Ilji Miecznikowa 1, 02-096 Warsaw, Poland; a.girstun@uw.edu.pl (A.G.); j.trzcinska-da@uw.edu.pl (J.T.-D.)

**Keywords:** necrotizing enterocolitis, inflammation mediators, adipose tissue-derived stem cells

## Abstract

**Background/Objectives**: Research on the roles of stem cells in necrotizing enterocolitis (NEC) has primarily focused on the effects of bone marrow- and amniotic fluid-derived stem cells in mitigating the clinical manifestations of the disease. However, the potential of adipose tissue-derived stem cells (ADSCs) remains unexplored in this context. The aim of this study was to evaluate the therapeutic potential of ADSC administration during the active inflammatory phase of NEC, with a specific focus on reducing the levels of the inflammatory cytokines IL-1 and IL-6. **Methods**: A self-modified hypoxia–hypothermia–formula feeding rat NEC model was employed. A total of 117 rat pups were divided into two groups: a treatment group (NEC-ADSC, n = 55) and a control group (NEC-PLCB (placebo), n = 62). In the NEC-ADSC group, ADSCs were administered intraperitoneally 24 h into the NEC protocol. After 72 h, bowel and fluid samples were collected for analysis. **Results**: The analysis revealed no significant effect on NEC histopathology (*p* = 0.347) or on the levels of IL-1 and IL-6 (*p* = 0.119 and *p* = 0.414, respectively). **Conclusions**: The administration of adipose tissue-derived stem cells after the onset of necrotizing enterocolitis does not reduce the levels of inflammatory cytokines IL-1 and IL-6, nor does it influence the histopathological outcomes of the disease in the rat model. Further research is needed to explore the potential therapeutic role of adipose tissue-derived stem cells in the treatment of necrotizing enterocolitis.

## 1. Introduction

### 1.1. Necrotizing Enterocolitis (NEC)

Necrotizing enterocolitis (NEC) is a neonatal condition characterized by widespread inflammation and necrosis of the bowel wall [1]. NEC predominantly impacts premature infants, with the risk increasing as birth weight decreases. The highest morbidity and mortality rates are observed in neonates weighing less than 1000 g. The overall incidence of NEC is approximately 1 in 1000 live births, rising to 20% among infants with very low birth weights [2]. Mortality rates are estimated to range from 15% to 30%, with survival inversely correlated with gestational age and birth weight [2]. Although necrotizing enterocolitis remains the leading cause of surgery in neonates, its exact mechanism of development is not yet fully understood. Several key mechanisms are thought to contribute to the disease, including hypoxia, reduced intestinal blood flow, and bacterial colonization [3,4]. The primary risk factors contributing to NEC are very low birth weight, early formula feeding, intestinal dysbiosis, prematurity, hypoxia, and congenital heart defects. Furthermore, maternal factors such as gestational infections, metabolic conditions, cocaine use, and hypoxia may also increase the risk [1,2,4]. The management of NEC encompasses both medical and surgical approaches, with bowel perforation serving as a primary indication for surgical intervention [2,4,5]. Conservative management generally includes the administration of broad-spectrum antibiotics, discontinuation of oral feeding, and gastrointestinal decompression [2,5]. Surgical approaches vary based on the patient’s clinical status and the extent of bowel necrosis. Regarding prevention, only the protective effect of maternal breast milk is strongly supported by evidence [1,4]. NEC research relies on various animal models, including mice, rats, and pigs [4,6,7]. While rat models are valuable for assessing the safety and effectiveness of new therapies, mouse models offer the added advantage of genetic modification [4,6,7]. Piglet models, though costly, are the closest in gastrointestinal structure to humans, making them particularly useful in NEC research [4,6].

### 1.2. Stem Cells

A stem cell (SC) is an unspecialized (or low-specialized) cell that has the capabilities of renewal, division, and giving rise to specialized cell types [8]. Among the two types—namely, embryonic and somatic stem cells—the latter do not raise ethical concerns when considered in scientific research. The capacities of SCs to self-renew, differentiate, inhibit apoptosis, and limit inflammation make the potential use of SCs in the prophylaxis of NEC reasonable.

### 1.3. Cytokines in NEC

The cytokine pathway is crucial in the development of necrotizing enterocolitis (NEC) [9]. The initial injury due to hypoxia and hypothermia results in damage to the intestinal mucosa. This, in combination with artificial feeding and microbial colonization of the underdeveloped mucosal lining, triggers the release of multiple inflammatory mediators, including platelet-activating factor (PAF) and tumor necrosis factor-alpha (TNF-α). Bacterial products such as lipopolysaccharide (LPS) further hinder the mucosal regenerative ability through suppressing enterocyte migration [9,10]. This leads to a disruption of the mucosal barrier, facilitating bacterial translocation, vasoconstriction, and activation of the inflammatory cascade, ultimately leading to ischemia and subsequent necrosis of the wall of the intestine [9,10]. NEC-affected human tissue samples demonstrate elevated mRNA expression of interleukin (IL)-1B, IL-8/CXCL8, and TNF [11]. In a microarray analysis performed by MohanKumar et al., NEC bowel samples showed increased expression of numerous cytokines, including IL-1A, IL-1B, IL-6, IL-10, TNF, hepatocyte growth factor (HGF), and vascular endothelial growth factor (VEGF)-A, when compared to normal intestine [11]. Additionally, plasma concentrations of cytokines, including IL-6, IL-8/CXCL8, and IL-10, were increased in patients with NEC [11].

Interleukin 1 (IL-1), which is produced by macrophages, consists of two forms: IL-1α (a cell-associated protein) and IL-1β (a secreted cytokine). Stimulated by tumor necrosis factor-alpha (TNF-α), IL-1 amplifies the inflammatory response, contributing to fever, enhanced endothelial adhesion of leukocytes, activation of phagocytes, and stimulation of lymphocytes. Meanwhile, IL-1β is involved in increasing neutrophil recruitment to sites of inflammation through inducing the IL-8 gene. IL-1 is a key mediator in systemic inflammatory response syndrome (SIRS), a condition that can progress to sepsis and multiple organ failure [9].

Interleukin 6 secretion is enhanced by various additional pro-inflammatory mediators, including TNF-α and IL-1 [8,10]. IL-6 stimulates lymphocyte activation, facilitates antibody production by B cells, and supports the differentiation of cytotoxic T cells [9,10]. In the context of systemic inflammatory response syndrome (SIRS) and sepsis, enterocytes exhibit elevated secretion of IL-6, which is also presented by the intestinal endothelium, macrophages, and helper T cells [9]. Its production is further amplified by bacteria, endotoxins, and various cytokines [9]. Acute-phase reactants such as C-reactive protein are also stimulated by IL-6 [9].

### 1.4. Adipose-Derived Stem Cells and NEC

Stem cells can be derived from various sources and are present in varying quantities across different tissues, including the liver, kidney, skin, bone marrow, adipose tissue, placenta, dental tissue, amniotic fluid, amnion, umbilical cord blood, and various fetal tissues [12,13,14]. Adipose tissue has emerged as a significant source of MSCs, due to its unrestricted donor availability and the minimal risk of side effects associated with cell harvesting [15]. Adipose-derived stem cells (ADSCs) exhibit surface markers and differentiation potential comparable to bone marrow-derived stem cells (BMDSCs). Under suitable conditions, they can differentiate into cell types originating from all three germ layers [16]. A single gram of human adipose tissue can yield approximately 5 × 10⁹ stem cells, which is 500 times more than the number of MSCs obtained from an equivalent amount of bone marrow [17]. They show strong proliferative ability and retain their multi-differentiation potential after many passages [15]. Research has shown that mouse ADSCs are capable of secreting a variety of angiogenic growth factors and cytokines [18]. A limited amount of data gathered from research concerning bone marrow- and amniotic fluid-derived mesenchymal stem cells (BMDSCs and AFDSCs, respectively) has demonstrated a significant reduction in the incidence and severity of NEC in animal models [13,19,20,21,22].

Stem cells primarily modulate the immune system through two key mechanisms: direct cell-to-cell interactions and indirect actions mediated by the secretion of soluble factors, growth factors, and extracellular vesicles [23]. Immunoregulation through direct interaction with immune cells is facilitated by the paracrine secretion of cytokines, growth factors, and other soluble mediators [23]. Adipose-derived stem cells (ADSCs) have demonstrated the ability to affect various immune cells, including T cells, B cells, and macrophages [24,25]. They facilitate the formation of new regulatory T cells [26,27], promote macrophage polarization toward the M2 phenotype, reduce the expression of pro-inflammatory cytokines (e.g., TNF, IL-1, IFN, and IL-12), and enhance the production of anti-inflammatory cytokines, including IL-10 [28,29,30]. ADSCs secrete soluble factors and extracellular vesicles, including exosomes and microvesicles [31,32]. Their primary mechanism for immune modulation is thought to be paracrine [8,33]. The soluble mediators include a range of pro- and anti-inflammatory cytokines, along with factors such as vascular endothelial growth factor (VEGF), insulin-like growth factor (IGF-1), and granulocyte colony-stimulating factor (G-CSF) [34]. Exosomes derived from ADSCs are lipid-membrane nanovesicles (30–100 nm) containing proteins, lipids, DNA, mRNAs, microRNAs, tRNA, and non-coding RNAs [23,35,36]. The results of a study by Lai et al. suggested that MSC-derived exosomes from different sources share similar biological properties [33]. These exosomes exert therapeutic effects through paracrine signaling, releasing their contents into the cytoplasm of different cells. They appear to influence the immune system in a manner similar to ADSCs, although the exact mechanisms remain unknown [23]. Experimentally, exosomes have shown clinical potential via enhancing wound healing through collagen synthesis [37,38], reducing inflammatory cell infiltration in dermatitis [39], and lowering cardiomyocyte apoptosis due to oxidative stress [40].

### 1.5. Aim of the Study

The aim of this study was to assess the curative potential of ADSC administration during the ongoing inflammatory process in NEC. Intraperitoneal injection of ADSCs during the disease was expected to lower the inflammatory response, resulting in a decreased incidence of NEC and reduced levels of the inflammatory cytokines IL-1 and IL-6.

## 2. Materials and Methods

Approval was obtained from the Local Ethical Committee for Animal Experiments (WAW2/093/2021). We employed a self-modified version of the hypoxia–hypothermia–formula feeding rat NEC model to evaluate the impact of intraperitoneal administration of ADSCs after disease onset on the inflammatory profile and histopathological features of NEC [4].

A sample size calculation was conducted to determine the number of subjects required to demonstrate the study’s primary endpoint. This estimation was based on data from a pilot study and relevant research [4]. The primary endpoint was defined as the proportion of animals developing NEC within each group, with a planned comparison between the experimental and control groups. Based on the assumptions, it was anticipated that the NEC incidence in the experimental group would be approximately 30% below that of the control group, where the expected incidence of necrotizing enterocolitis is around 75%. Using a chi-square test for comparison between groups, at a significance level of 0.05 and a statistical power of 70%, the necessary sample size per group was found to be 30 animals. Allowing for an estimated 50% dropout rate, the adjusted required sample size per group was approximately 60 animals.

During the experiment, animals were housed in individually ventilated cages, providing optimal maintenance conditions. The ambient temperature in both the cages and the room housing the animals was set at 22 °C (±2 °C), with humidity at 55% (±5%). A 12 h light/12 h dark cycle was observed. The ventilation system supplied 20 air exchanges per hour in the room, while air in the animal cages was exchanged 75 times per hour. Continuous monitoring of temperature and humidity was conducted in both the rooms and individually ventilated cages.

Newborn *SPRD* rat pups in the experimental group (NEC-ADSC, n = 55) received an intraperitoneal injection of 6 × 10^5^ labeled cells suspended in 50 μL of PBS, administered 24 h after the initiation of the NEC model. Animals in the control group (NEC-PLCB (placebo), n = 62) were administered 50 μL of PBS. This method of administration and count of cells have been proven to be effective in previous works by other authors (see, e.g., McCulloh et al. [19,21]).

### 2.1. NEC Induction

The animals were included in the NEC induction protocol [4]. The animals were exposed to a 100% nitrogen atmosphere for 60 s in IVS cages, followed by the restoration of normal conditions after one minute. The cages were then placed in a cooling device set to 4 °C for 10 min, after which normal conditions were reinstated. The animals were fed a tolerated volume of commercially available formula milk using a 0.5 mm pipette. After a set period of 72 h (or earlier in cases of spontaneous death, provided it was no less than 24 h), bowel fragments—at least three samples from macroscopically affected regions of the duodenum, small intestine, and large intestine—were collected. These samples were preserved in 10% neutral buffered formalin for subsequent analysis.

### 2.2. Stem Cells

**Figure 1:**
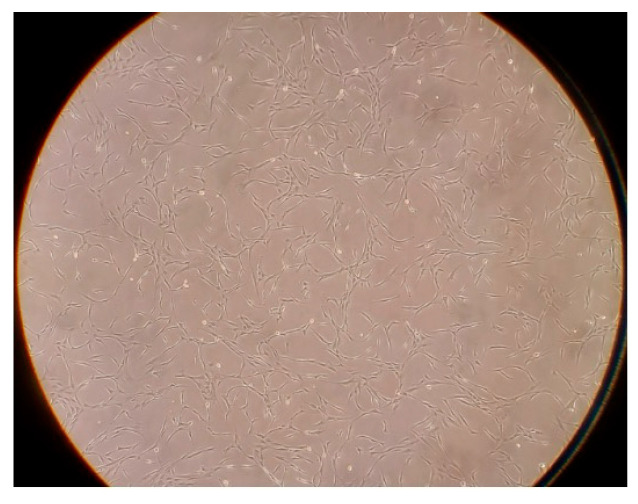
Stem cell culture observed under a light microscope (Olympus CK30, Olympus Optical Co., Ltd., Tokyo, Japan WIK10x20L). Source: author’s materials.

StemPro Human Adipose-Derived Stem Cells (Gibco, Thermo Fisher Scientific, Life Sciences Solutions, #R7788110, Carlsbad, CA, USA) were cultured in MesenPRO RS Basal Medium supplemented with MesenPRO RS Growth Supplement (included with the ADSC cells by Gibco), 2 mM L-glutamine, 100 μg/mL streptomycin, and 100 U/mL penicillin. The cells were cultured at 37 °C in a humidified environment containing 5% CO_2_ (Figure 1). For experimental use, cells at passage levels below 5 were selected. When the cells reached 80% confluency, they were rinsed with PBS and detached from the culture vessels using Accutase^®^ solution. The detached cells were collected, centrifuged at 250× *g* for 5 min at room temperature, counted, washed with PBS, and centrifuged again. Finally, the cells were resuspended in PBS and divided into portions of 6 × 10^5^ cells per 50 µL.

### 2.3. Immunohistochemistry—Engraftment Analysis

**Figure 2:**
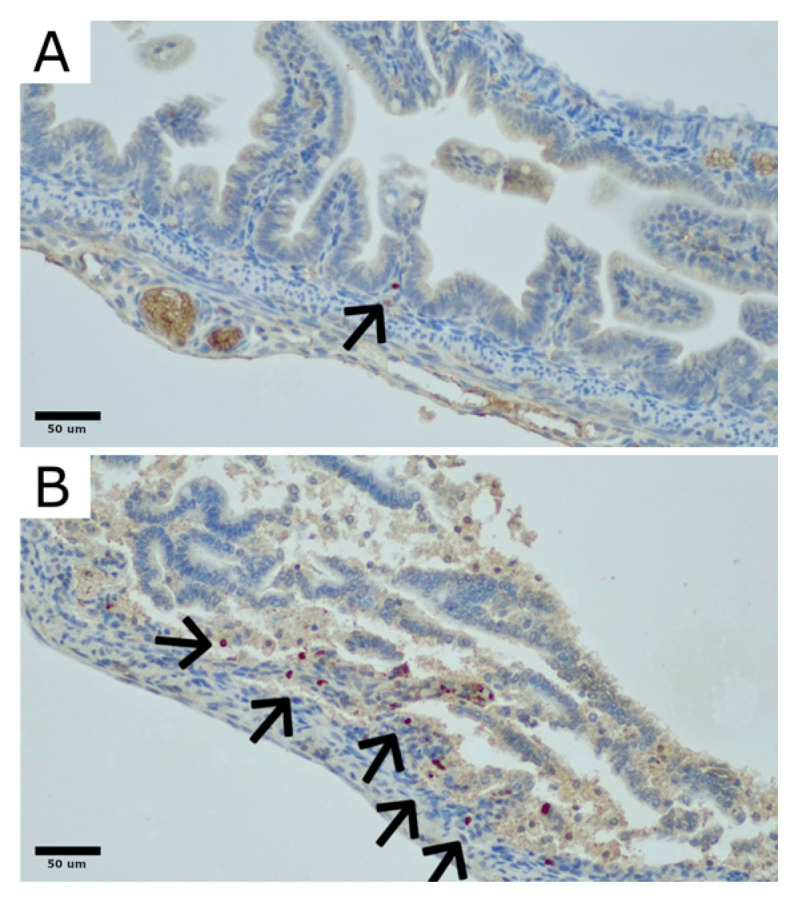
Examples of CD90-positive cell distribution in the rat bowel: (**A**). Section of colon showing partial villous atrophy with only a single CD90-positive cell (indicated by an arrow). (**B**). Section of colon exhibiting sloughing and necrosis of the upper parts of atrophic villi, with scattered CD90-positive cells present in the mucosa and submucosa (indicated by arrows). Original magnification: 400×. Microphotographs were captured using the OPTIKA LITEView software (OPTIKA, Version: Windows x64 2.1.24744.20240303; OPTIKA, Ponteranica, Italy). Source: author’s materials.

To evaluate the presence of CD90-positive cells, sections of rat bowel were stained with an anti-CD90 polyclonal rabbit antibody (LifeSpan Biosciences, Lynnwood, WA, USA), in accordance with the manufacturer’s protocol (Figure 2). Areas with the highest concentration of CD90-positive cells (“hot spots”) were identified at 100× magnification. Within these regions, the quantity and distribution of CD90-positive cells were analyzed at 200× magnification and reported as a percentage of the total cell population in the bowel mucosa and submucosa.

### 2.4. Histopathology

**Figure 3:**
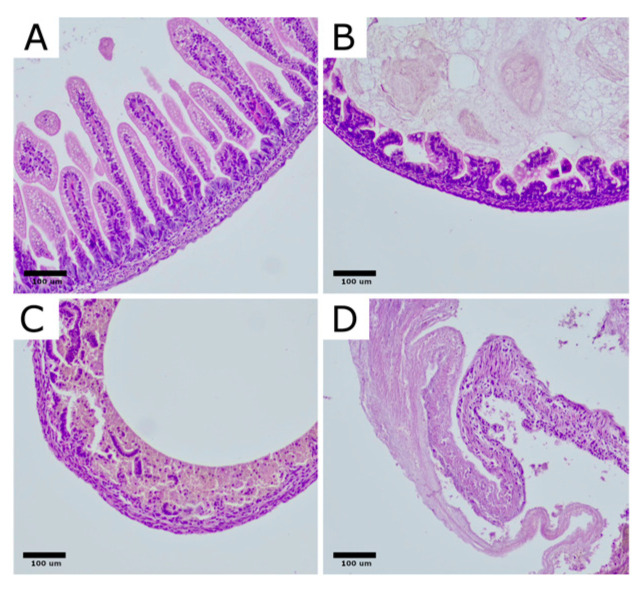
Histological alterations in the intestinal architecture of rats with NEC are depicted. Rat bowel sections stained with H&E demonstrate representative samples corresponding to each morphological severity score: (**A**). normal ileum, NEC score 0; (**B**). NEC score 1, partial villous atrophy; (**C**). NEC score 2, sloughing and/or necrosis of upper parts of atrophic villi; (**D**). NEC score 3, total loss of villi and necrosis of the intestinal wall. Original magnification 200×. Microphotographs were captured using the OPTIKA LITEView software (OPTIKA, Version: Windows x64 2.1.24744.20240303; OPTIKA, Ponteranica, Italy). Source: author’s materials.

Each specimen was preserved in 10% neutral buffered formalin. Following fixation, the samples were embedded in paraffin, sectioned into 3 µm thick slices, and stained with hematoxylin and eosin (H&E) for morphological evaluation. An initial examination was conducted at low magnification (40× scanning), in order to evaluate the overall structure of the rat bowel. Further analysis was performed at 200× and 400× magnifications, using a semiquantitative four-tier NEC severity grading scale: grade 0, no changes observed in the bowel wall; grade 1, partial villous atrophy; grade 2, sloughing and/or necrosis of the epithelium in the upper portions of atrophic villi; and grade 3, complete loss of villi and necrosis of the intestinal wall [4,41] (Figure 3).

### 2.5. ELISA Analysis—Cytokine Concentrations

IL-1 and IL-6 levels in peritoneal fluid were quantified using a commercial ELISA test kit from R&D Systems (Minneapolis, MN, USA) following the manufacturer’s instructions. The procedure began with the addition of 50 μL of assay diluent to each well, followed by 50 μL of standard, control, or sample. The plate was gently mixed, covered, and incubated at room temperature for 2 h. Once incubation was complete, the wells were aspirated and washed five times with wash buffer, ensuring complete removal of liquid each time. Next, 100 μL of IL-6 or IL-1 conjugate was added to each well. The plate was then covered and incubated for another 2 h at room temperature. Following a second wash, 100 μL of substrate solution was introduced, and the plate was shielded from light and incubated for 30 min. Finally, 100 μL of stop solution was added, and the optical density was recorded at 450 nm within 30 min with wavelength correction applied at 540. For each sample in the ELISA test, three measurements were performed.

### 2.6. Statistical Analysis

The analyses were performed utilizing R software (R Core Team, 2023, https://www.R-project.org/, accessed on 15 December 2024), and visualization of the results was performed using the “ggplot2” graphics package (Wickham, 2016, https://ggplot2.tidyverse.org, accessed on 15 December 2024). To assess the impacts of the Group variable on IL1, IL6, and CD90, a one-way analysis of comparisons for independent samples was conducted. Given the non-parametric nature of the data, the Kruskal–Wallis test was used. The association between the NEC and Group variables was analyzed using a chi-square test. However, due to the presence of expected values below 5, statistical significance was adjusted with Fisher’s exact test.

Language improvement for this manuscript was supported by the use of ChatGPT 4.0, an AI language model developed by OpenAI. The AI was utilized solely for enhancing graphs and the language quality of the text and was not involved in generating data, designing the study, or writing its content.

## 3. Results

### 3.1. Engraftment

To assess the level of engraftment into the bowel tissue, injected human adipose-derived stem cells were highlighted by means of immunofluorescence staining with anti-CD90 antibodies using the streptavidin–biotin method.

**Figure 4:**
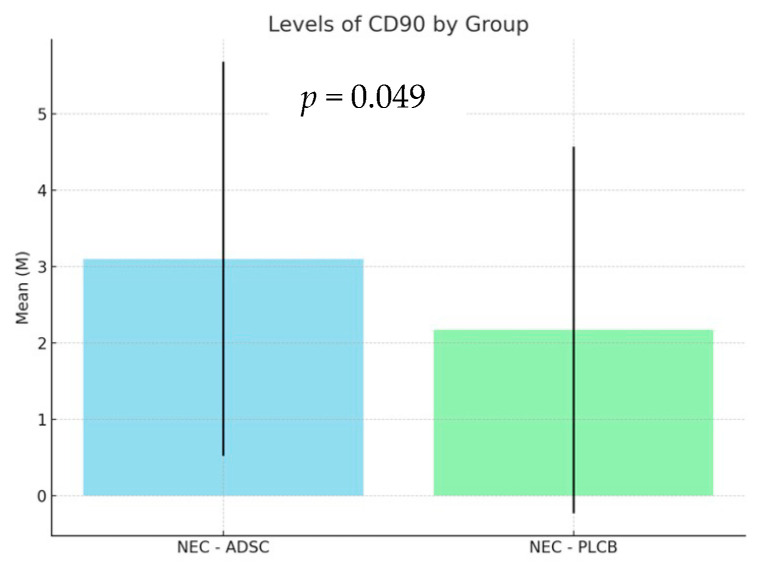
Levels of CD90 antibodies, expressed as percentage of the total cells present in the bowel mucosa and submucosa.

Due to the non-parametric nature of the analyzed data, a Kruskal–Wallis test was performed, revealing a significant effect of the Group variable on the CD90 variable results [H(2) = 6.04; *p* = 0.049; η^2^H = 0.02]. The levels of CD90 antibodies in the NEC-ADSC and NEC-PLCB groups are summarized in Table 1 and Figure 4 below.

Levels of engraftment were significantly higher in the NEC-ADSC group (*p* = 0.049).

### 3.2. Histopathology

For histopathological evaluation, a total of 117 specimens were initially preserved in 10% neutral buffered formalin. However, due to technical issues, 2 samples from the NEC-PLCB group could not be processed, leaving a total of 115 samples for examination. The preserved tissues were subsequently embedded in paraffin, cut into 3 µm thick sections, and stained with hematoxylin and eosin (H&E) for routine morphological analysis [4,41].

Further analysis was performed at 200× and 400× magnifications, using a semiquantitative four-tier NEC severity grading scale: grade 0, no changes observed in the bowel wall; grade 1, partial villous atrophy; grade 2, sloughing and/or necrosis of the epithelium in the upper portions of atrophic villi; and grade 3, complete loss of villi and necrosis of the intestinal wall [4,41].

The findings from the histopathological analysis are summarized in Table 2 below.

**Figure 5:**
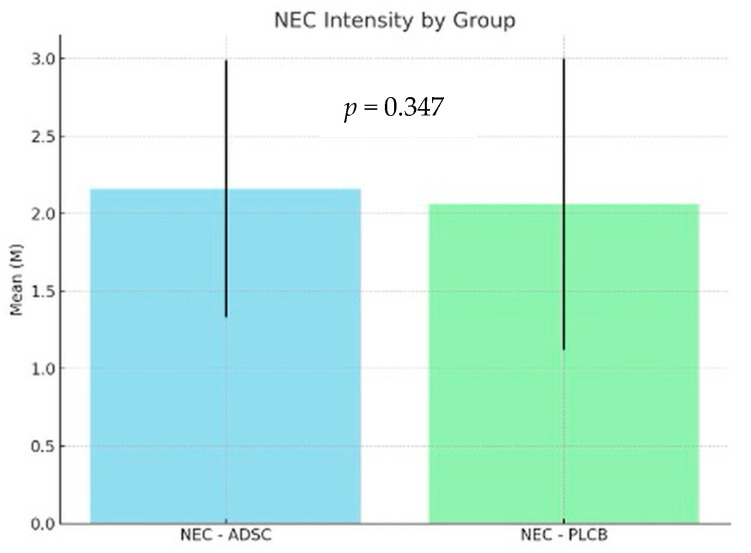
NEC intensity—the mean value of severity grade of all samples within each group of the NEC variable.

Given the non-parametric nature of the data, a Kruskal–Wallis test was performed to evaluate the histopathological scoring. The analysis revealed no significant effect of the Group variable on the NEC variable [H(2) = 2.12; *p* = 0.347; η^2^H = 0.00]. The intensity, defined as the mean severity grade of all samples within each group of the NEC variable, was comparable across the analyzed groups. The results are detailed in Table 3 and Figure 5 below.

**Figure 6:**
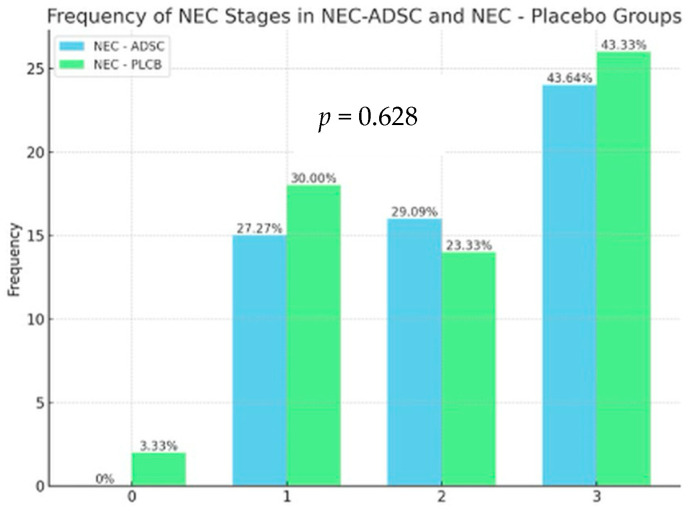
Frequency of NEC stages in groups.

To examine the relationship between the NEC and Group variables, a chi-square test was performed. Since expected values lower than 5 were observed, Fisher’s exact test was used to adjust for significance. The analysis indicated no significant association between the NEC and Group variables [χ^2^(6) = 4.36; *p* = 0.628]. The distribution of NEC variable occurrences was consistent across the various levels of the Group variable. The results are presented in Figure 6.

### 3.3. Cytokine Concentrations

The levels of IL-1 and IL-6 in the NEC-ADSC and NEC-PLCB groups (n = 117) were measured using commercial ELISA kits. The detailed results are provided in Table 4 and Table 5 below.

**Figure 7:**
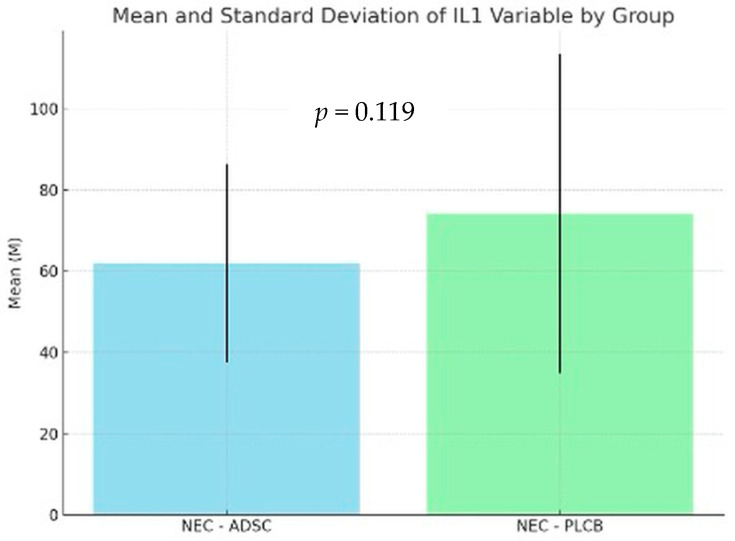
Levels of IL-1 within groups (pg/mL).

**Figure 8:**
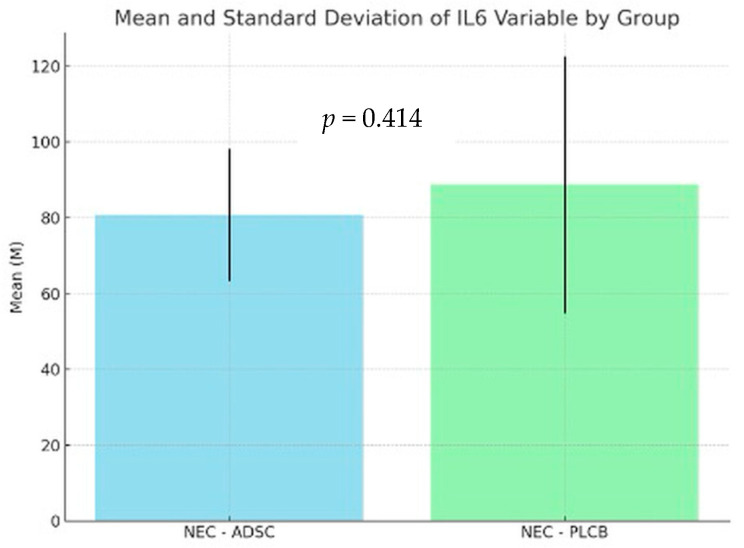
Levels of IL-6 within groups (pg/mL).

Levene’s test indicated that the assumption of equal variances was violated in the tested groups. As a result, Welch’s correction for unequal variances was applied. The analysis for IL-1 levels showed no statistically significant difference between the NEC-PLCB and NEC-ADSC groups (*p* = 0.119). The average intensity of the IL1 variable in the NEC-PLCB group was statistically similar when compared to the results in the NEC-ADSC group, with the intensity of these results being M = 74.18; SD = 39.31 vs. M = 61.9; SD = 24.38, respectively. For IL-6, the analysis revealed no statistically significant difference between the NEC-PLCB and NEC-ADSC groups (*p* = 0.414). The average intensity of the IL-6 variable in the NEC-PLCB group was statistically similar when compared to the results in the NEC-ADSC group, with the intensity of these results being M = 88.67; SD = 35.95 vs. M = 80.74; SD = 17.46, respectively. The results are presented in Figure 7 and Figure 8 below.

### 3.4. Summary of the Results

The levels of the CD90 molecule were significantly elevated in the NEC-ADSC group compared to the NEC-PLCB group.

The NEC intensity—defined as the mean severity grade of all samples within each group—was comparable between the NEC-ADSC and NEC-PLCB groups.

The frequencies of NEC stages (by the scoring scale) were similar in the NEC-ADSC and NEC-PLCB groups.

The levels of the IL-1 and IL-6 variables in the NEC-PLCB group were statistically similar, when compared to the results in the NEC-ADSC group.

## 4. Discussion

We hypothesized that stem cells administered after the onset of NEC in the rat model would reduce the levels of the inflammatory cytokines IL-1 and IL-6, compared to the NEC group receiving only saline injections, which would suggest a possible future role in the treatment process of NEC. The onset of NEC initiates a significant cytokine response at both the mucosal and systemic levels, including IL1 and IL6 elevation, among many other factors, being consistent with the overall picture of necrotizing enterocolitis [9,11]. To the contrary, stem cells have shown anti-inflammatory potential through lowering the levels of inflammatory cytokines in various (mostly paracrine and cell-to-cell) mechanisms [42,43,44]. Yang et al. demonstrated that extracellular vesicles derived from bone marrow mesenchymal stem cells effectively reduced the levels of inflammatory cytokines in a rat model of colon inflammation [42]. Ocansey et al. demonstrated the effects of MSCs on lowering the inflammatory cytokines levels in the setting of inflammatory bowel diseases in detail [43]. To demonstrate this effect, a self-modified hypoxia–hypothermia NEC model was utilized [4]. This model has previously been utilized to demonstrate the prophylactic effect of maternal milk in reducing the incidence of NEC [4]. We did not observe a statistically significant difference between the ADSC and placebo groups in terms of the levels of IL-1 and IL-6. While a trend toward reduced cytokine levels was apparent, no definitive conclusions can be drawn regarding the effect of ADSCs. To the best of our knowledge, this is the first study to evaluate the use of ADSCs in the context of necrotizing enterocolitis. Other MSCs have shown effectiveness in the NEC environment, as described by McCulloch et al. [21]. Amniotic fluid- and bone marrow-derived stem cells were tested for their impact on the NEC histopathology in a rat model of NEC [21]. All tested stem cell types demonstrated an equivalent ability to reduce the severity of necrotizing enterocolitis [21]. This aligns with the findings of Zani et al., where amniotic fluid stem cells integrated into the bowel wall, improving survival and clinical outcomes in rats. Their research demonstrated a reduction in NEC incidence and macroscopic gut damage, enhanced intestinal function, reduced bowel inflammation, increased enterocyte proliferation, and decreased apoptosis. These effects were mediated through the modulation of stromal cells expressing cyclooxygenase-2 in the lamina propria [22].

Adipose-derived stem cells are mesenchymal stem cells located within fat tissue. They are easily available using a liposuction technique, do not pose ethical dilemmas in the context of clinical application and research, and share properties with other MSCs. An increasing body of research has highlighted that ADSCs possess immunomodulatory, anti-inflammatory, and proangiogenic properties [23,45]. The only study to date reporting on the effect of adipose tissue in the setting of necrotizing enterocolitis was conducted by Mimatsu et al. [46], who showed that the administration of differentiated fat cells, and not adipose-derived stem cells, improved survival rates and facilitated the repair of damaged intestinal tissues in NEC, potentially through normalizing the expression of fatty acid-related proteins, reducing inflammation, and lowering the levels of IL-1 and IL-6 [46]. The hypothesis regarding the contrary results of our study might be that the moment of injection of the cells into the peritoneal cavity might be crucial for the ADSCs to present an effect. Typically, the stem cells are injected at the beginning of the experiment, together with the initiation of the NEC model [19,22]. One can discuss whether researchers can measure the prophylactic or curative potential of the cells by doing so. On one hand, engraftment of the cells will be simultaneous to the NEC induction; on the other hand, stem cells have the ability to impose immediate action via their paracrine properties. Our goal was to test the curative potential in the ongoing NEC process. Therefore, we decided to inject the cells 24 h after the initiation of the model. The levels of CD90 antibody used for the identification of the engraftment of stem cells differed between groups, which is consistent with the work of other researchers [47]. The potential to engraft into injured tissues is greater than into the healthy bowel. Therefore, stem cells injected during the ongoing high-level inflammatory process will have a strong tendency to engraft. Again, the moment of injection during an ongoing inflammatory process, on one hand, enables the movement of stem cells to the injured bowel while, on the other hand, there might not be enough time for the cells to engraft before the bowel wall turns necrotic.

Most experimental works on the effectiveness of MSCs in the setting of NEC have involved histopathological assessment of the bowel. In the works by McCulloch et al., the application of many kinds of SCs in experimental rat necrotizing enterocolitis limited the incidence and severity of experimental NEC, as evaluated through histopathological analysis [19,21]. Our hypothesis was that reducing the inflammatory reaction through the administration of ADSCs affects the histopathological picture of NEC and decreases the severity of the disease. No statistically significant differences were observed between the groups regarding the severity of NEC or the frequency of its different stages. As the model of NEC utilized in the experiment was a modification of a well-established hypoxia–hypothermia–formula feeding model of necrotizing enterocolitis in rats via single exposure to NEC-causing factors [4], we assumed that either the NEC-inducing factors in our model were insufficient to reveal the impact of ADSC injection on histopathology, or the timing of the ADSC injection was too delayed for the stem cells to exert a measurable effect. The effect of the engrafted cells could be limited by the level of morphological changes caused by the generalized inflammation, which can be compared to the clinical picture of end-stage NEC. Due to the aforementioned reasons, it is possible that the effectiveness of ADSCs used as curative agents is highly dependent on the moment of injection.

The clinical application and experimental study of MSCs in the neonatal setting have been widely explored over the last decade regarding many indications, including brain injury, heart diseases, and eye, lung, and gut diseases [48]. Phase I clinical trials conducted by various groups have demonstrated the beneficial effects of stem cell administration in the context of conditions such as bronchopulmonary dysplasia. These include reducing inflammatory cytokine levels in the trachea, decreasing disease severity, and lowering the need for supplemental oxygen [48]. In addition, retinopathy of prematurity requiring surgical intervention occurred less frequently in the MSC-treated group, compared to historical controls [48]. MSC treatment also reduced the risk of neurodevelopmental morbidities [48]. To date, a single clinical application of umbilical cord-derived MSCs in the treatment of necrotizing enterocolitis has been reported by Akduman et al. [49]. One dose of 1 × 10⁷ allogeneic umbilical cord-derived mesenchymal stem cells (UC-MSCs) was transfused without complications 4 days after colectomy with 60 cm bowel resection in a full-term 3.350 kg patient, who developed NEC due to repeated supraventricular tachycardia. An improvement in ultrasonography was observed on the third day post-transplantation, enteral nutrition was reinstated on day 8, and the jejunal stoma was closed 46 days after the transplantation [49]. Although not properly designed to verify the correlation of the application of SCs with the result, this case report has to be noted in terms of the safety of the method at minimum. Given the effect of ADSC administration after the onset of NEC observed in our study, it can be hypothesized that it will be dependent on the moment of injection of stem cells, which could affect the possibility to engraft into the tissues. In contrast, the effects of MSC administration even before engraftment have been demonstrated by Pisano et al. [47], who explored the roles of stem cells, heparin-binding epidermal growth factor-like growth factor, and stem cell-derived exosomes in preventing the disease [47]. As the observed effects occurred prior to the potential engraftment of stem cells into bowel tissue, they proposed that the primary mechanism by which stem cell administration prevents NEC is not through engraftment but, rather, via the paracrine or endocrine secretion of factors, exosomes, or vesicles [47]. Still, the intensity of NEC and the inflammatory cascade at the moment of cells’ administration could, in our opinion, highly affect the effectiveness of the treatment.

The limitations of this study involve the arbitrary selection of the moment of application of the stem cells. Knowing the results of the experiment, it could be worth determining whether different moments of ADSC application could lead to different effects. Furthermore, we were hypothesizing what could have caused the minimal impact on histopathology in our experimental setting. The tendency observed in the levels of inflammatory cytokines—even if not statistically significant—should, in our opinion, affect the histopathological results. We assumed that one of the reasons for the low heterogeneity of the results could be the tendency of the model to present the higher stages of necrotizing enterocolitis; in other words, the sensitivity of the model used may not be sufficient to elucidate the treatment effects. Our model has previously been utilized to demonstrate the protective effect of maternal milk in reducing the incidence of NEC [4]; however, the established hypothermia–hypoxia–formula feeding model utilized by McCulloh et al., among others, to demonstrate the impact of SCs on the histopathological picture of NEC could be tested for, and may be more effective in demonstrating the curative effects of SCs, as it was effective in proving the prophylactic effects of the SCs [19,21]. Also, a way to differentiate between the effects of ADSCs and the insufficient model sensitivity could be parallel testing of different kinds of MSCs using our modified model—like in the works of McCulloch [21]. Knowing the results of the present experiment, we have re-evaluated the engraftment assessment. To draw more certain conclusions on the effects of ADSCs, engraftment should probably be assessed using more than one method; for example, through including measurements of the CD44 molecule. On the other hand, the Ki67 molecule could help in extracting the levels of proliferation to better assess the viability of the engrafted cells. Additionally, our group has designed another study to evaluate the effects of ADSCs administered prior to the onset of NEC, with the aim of assessing their prophylactic potential in controlling inflammation [41].

## 5. Conclusions

The administration of adipose tissue-derived stem cells after the onset of necrotizing enterocolitis did not reduce the levels of inflammatory cytokines IL-1 and IL-6, nor did it influence the histopathological outcomes of the disease in a rat model of NEC.

Further research is needed to explore the potential therapeutic role of adipose tissue-derived stem cells in the treatment of necrotizing enterocolitis.

## Figures and Tables

**Table 1:** Levels of CD90 antibodies, expressed as percentage of the total cells present in the bowel mucosa and submucosa. NEC-ADSC—treatment group, NEC-PLCB—control group, PLCB—placebo.

Group	n	Min	Max	M	SD
NEC-ADSC	55	0	10	3.10	2.58
NEC-PLCB	60	0	10	2.17	2.40

**Table 2:** Histopathological results, in terms of the number of samples in each severity grade.

NEC Score	NEC-ADSC	NEC-PLCB
0	0 (0.00%)	2 (3.33%)
1	15 (27.27%)	18 (30.00%)
2	16 (29.09%)	14 (23.33%)
3	24 (43.64%)	26 (43.33%)
Total	55 (100.00%)	60 (100.00%)

**Table 3:** NEC intensity—the mean value of severity grade of all samples within each group of the NEC variable.

Group	n	Min	Max	M	SD
NEC-ADSC	55	1	3	2.16	0.83
NEC-PLCB	60	0	3	2.06	0.94

**Table 4:** Levels of IL-1 within groups (pg/mL).

Group	n	Min	Max	M	SD
NEC-ADSC	55	5	112.19	61.90	24.38
NEC-PLCB	62	10	189.49	74.18	39.31

**Table 5:** Levels of IL-6 within groups (pg/mL).

Group	n	Min	Max	M	SD
NEC-ADSC	55	41.38	120.42	80.74	17.46
NEC-PLCB	62	25.30	180.40	88.67	33.95

## Data Availability

All data are available by request from authors.
